# Evaluation of Soil Amelioration Effects of Different Afforestation Measures in Alpine Sandy Land: A Case Study of the Gonghe Basin

**DOI:** 10.3390/microorganisms13122860

**Published:** 2025-12-16

**Authors:** Shaobo Du, Huichun Xie, Gaosen Zhang, Feng Qiao, Tianyue Zhao, Guigong Geng, Chongyi E

**Affiliations:** 1 College of Geographical Sciences, Qinghai Normal University, Xining 810008, China; wo827288809@163.com; 2 Qilian Mountain Southern Slope Forest Ecosystem Research Station, Huzhu 810500, China; qiaofnm@163.com; 3 Key Lab of Medicinal Animal and Plant Resources of Qinghai-Tibetan Plateau in Qinghai Province, Qinghai Normal University, Xining 810008, China; 4 College of Life Sciences, Qinghai Normal University, Xining 810008, China; xiehuichun1982@163.com; 5 Key Laboratory of Extreme Environmental Microbial Resources and Engineering, Northwest Institute of Eco-Environment and Resources, Chinese Academy of Sciences, Lanzhou 730000, China; gaosenzhang@hotmail.com; 6 Qinghai Academy of Agriculture and Forestry Sciences, Qinghai University, Xining 810016, China; genggg-298@163.com

**Keywords:** soil ecological indicators, sand land soil amelioration, alpine sandy land, sand control measures, Gonghe Basin, afforestation restoration

## Abstract

Desertification poses a severe challenge in China. Although long-term sand control measures have proven effective, the extensive and challenging nature of sandy land necessitates systematic research to identify optimal sand control measures for soil amelioration, thereby promoting ecological restoration in sandy areas. This study focused on the Gonghe Basin to assess the effectiveness of four 24-year afforestation treatments—*Salix cheilophila* + *Populus simonii*, *S. cheilophila*, *P. simonii* (YY), and *Caragana korshinskii*—compared to untreated mobile dunes. Surface soils (0–10 cm and 10–20 cm) were analyzed for physicochemical properties, enzyme activities, and bacterial community structure using Illumina high-throughput sequencing and PICRUSt2 functional prediction. All afforestation treatments significantly improved soil quality, increasing fine particle content, moisture, nutrients, enzyme activity, and microbial richness and diversity, especially in the topsoil. Bulk density and pH were notably reduced. Among the treatments, YY demonstrated the most substantial improvements. pH emerged as the primary factor influencing bacterial community structure, with enzyme activities also playing a significant role. Metabolism was the dominant functional category across all sites, while YY enhanced environmental information processing functions in the topsoil. Secondary functions showed high redundancy across treatments. These findings confirm that afforestation can effectively rehabilitate degraded alpine sandy soils, with the YY treatment offering the greatest benefits. The study provides a scientific basis for optimizing sand control measures and supports broader ecological restoration efforts in similar environments worldwide.

## 1. Introduction

Desertification is a land degradation process driven by sandy and loose surface conditions, arid climatic factors, and human activities. It currently represents one of the most important environmental threats globally [1,2]. China suffers severely from land desertification, with its northern regions being the most heavily impacted, due to specific climatic conditions and geographic factors. This has led to an extensive area of rapidly expanding sandy lands, making it a critical ecological issue that has garnered considerable attention. Consequently, related research and management practices have been actively promoted [3]. Sand control and prevention efforts have been ongoing in China for more than 70 years, leading to remarkable achievements in the ecological restoration of sandy areas [4]; however, the degree of land desertification remains severe, with the area of sandy land still accounting for 17.58% of the national land area [5]. The characteristics of sandy land include extensive area, broad distribution, substantial severity, and management challenges. This fundamental situation remains unchanged, with recurring risks of desertification in some managed areas. This underscores the importance and urgency of China’s ongoing efforts in sand control and prevention at this stage. This study focuses on the specific conditions of local sandy terrain to develop scientifically sound management strategies aimed at effectively mitigating the advance of desertification [6]. Land desertification involves the degradation of soil quality and limits plant survival. These processes, in turn, accelerate the land desertification process, thereby forming a vicious circle [7,8]. To select more appropriate sand control measures, various sand control measures should be scientifically evaluated based on differences in soil improvement effects. Among the current sand control measures, afforestation practices play a dominant role, and relevant studies [9,10,11,12,13] have indicated that these measures significantly increase the nutrient contents of organic matter and alkaline dissolved nitrogen in the surface layer of sandy soil and soil enzyme activities. Additionally, afforestation optimizes the structure of bacterial communities, thereby effectively ameliorating sandy soil. Soil physicochemical properties, enzyme activity, and bacterial community structure serve as critical indicators for evaluating soil quality in the context of assessing its improvement effects.

The Gonghe Basin is situated in the northeastern part of the Qinghai-Tibetan Plateau of China, with an average elevation of approximately 3200 m. It has a low temperature and dry climate and is highly impacted by desertification. It is characterized by a wide distribution of sandy land encompassing approximately 5573 km^2^, which is typical of alpine sandy land [14,15,16]. The ecological environment of the Gonghe Basin is fragile. Influenced by climatic factors and human activities, the degree of desertification has intensified, threatening local economic development and resource security. This highlights the necessity of implementing sand control and prevention measures in this region [17]. At present, the prevalent sand control measures in the alpine sands of the Gonghe Basin include afforestation measures and sand barrier deployment. Numerous scholars have evaluated the soil amelioration effects of afforestation practices [18,19]; however, these studies have predominantly focused on analyzing soil physical and chemical properties in isolation. Moreover, they have neglected to include the two key indicators of soil enzyme activity and bacterial community structure, which hampers a comprehensive understanding of the overall impact of afforestation measures on the soil system. The deployment of sand barriers can be divided into two types: living biological and mechanical sand barriers. Living biological sand barriers can rely on their own growth and development to improve soil quality [20,21]. However, these barriers predominantly consist of herbaceous plants, which are vulnerable to herbivory by gerbils and rabbits in sandy areas. This renders the deployment of biological living sand barriers unsuitable for long-term restoration. Conversely, mechanical sand barriers can remain in sandy environments for extended periods; however, their effectiveness in soil improvement is limited. Although mechanical sand barriers can be retained in the sandy environment for a long time, their efficacy at the soil improvement level is relatively limited [22]. This suggests that, in terms of soil amelioration effects, the comprehensive effectiveness of sand barrier installation is clearly weaker than afforestation measures. Therefore, in order to screen for sand control measures with optimal soil improvement effects in the alpine sandy areas of the Gonghe Basin, this study selected surface soil from four types of afforestation treatment areas established in 2000. The surface soil of the bare land of the mobile dune was used as the control. This study comprehensively assessed the effect of the four afforestation measures on the improvement of the surface layer of the sandy land soil. Specifically, we aimed to (1) determine and analyze the differences in physicochemical properties and enzyme activities of the surface soil in each treatment area and bare land; (2) analyze the bacterial community structure and function of the topsoil in each area and bare land using high-throughput sequencing and PICRUSt2 function prediction techniques; (3) elucidate the correlation between physical and chemical properties, enzyme activities, and bacterial community structure in the surface soil of each measure area and bare land.

## 2. Materials and Methods

### 2.1. Overview of the Study Area

**Figure 1:**
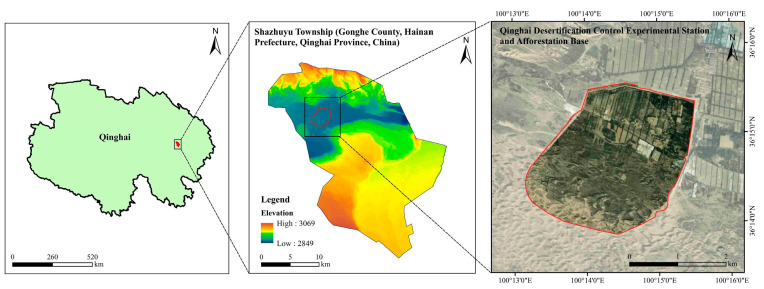
Overview of the study area.

The study area is located in Shazhuyu Township, Gonghe County, Hainan Prefecture, Qinghai Province, China, within an experimental forest field for sand control (100°25′ E, 36°24′ N, elevation 2880 m, Figure 1). This location forms part of the Gonghe Basin. The area has an alpine arid and semi-arid continental climate, an annual mean temperature of 2.0–3.3 °C, annual precipitation of 264 mm, and annual evapotranspiration of 1528–1937 mm. It experiences severe wind erosion of soils, with a prevailing wind direction from the northwest and west and an annual mean wind speed of 2.7 m/s [19]. Soil types include zonal calcareous and brown calcareous soils and non-zonal sandy, meadow, and swampy soils. The study area lacks a natural arboreal forest distribution, exhibiting a lower presence of herbaceous species and a higher prevalence of shrubby species, mainly *Caragana korshinskii* and *Artemisia desertorum.*

### 2.2. Sample Site Selection and Soil Sample Collection

Sample site selection and soil sample collection were performed in the study area in late July 2024 (during the peak growing season). Using the bare land of flowing dune (LD) as the control, four types of afforestation measures were selected: *Salix cheilophila* + *Populus simonii* plantation (WLYY00), *S. cheilophila* plantation (WL), *P. simonii* plantation (YY), and *C. korshinskii* plantation (NT00). All of these were established in 2000 (plant spacing 1.5 m × 1.5 m for all, Table 1). The soil types of the four afforestation practice areas were predominantly sandy and windy. The NT00 practice area afforestation method employed direct seeding afforestation, while the other three used seedling transplantation. The habitat conditions of all afforestation measure areas before establishment were fundamentally identical, as they were all situated in the flowing sand dunes on the alpine sandy land in the Gonghe Basin, which was representative of a uniform type of sand control measure. Six sample plots (i.e., six replicates), each measuring 50 m × 50 m, were randomly established in the inter-dune area of each sample plot, with a minimum distance of 50 m between each plot. Soil samples were collected at 0–10 cm and 10–20 cm depths in each sample plot using a five-point sampling method, resulting in a total of 60 soil samples [(6 soil samples from the 0–10 cm soil layer + 6 soil samples from the 10–20 cm soil layer) × 5 sample plots = 60 soil samples]. Each soil sample was divided into two parts: one was placed into 10 mL sterile centrifuge tubes and stored in liquid nitrogen for soil bacterial sequencing, while the other was used for assessing soil physical and chemical properties and enzyme activity.

### 2.3. Determination of Soil Physicochemical Properties and Enzyme Activities

Table 2 presents indicators of soil physical and chemical properties, as well as enzyme activities, along with their determination methods.

### 2.4. Extraction and Sequencing of 16S rDNA

Total bacterial DNA from soil samples was extracted using the MagaBio Soil Genomic DNA Purification Kit (Thermo Fisher Scientific, Shanghai, China) and subsequently analyzed via 1% agarose gel electrophoresis post-extraction. Polymerase chain reaction (PCR) amplification of the V3–V4 variable region of the 16S rRNA gene of the samples was performed using universal primers (338F and 806R) [29], while the reaction system and parameters were identical to those of Du et al. [30]. The reaction system is 20 μL: 5 × FastPfu Buffer, 4 μL; 2.5 mM dNTPs, 2 μL; Forward Primer (5 μM) and Reverse Primer (5 μM), 0.8 μL; FastPfu Polymerase, 0.4 μL; BSA, 0.2 μL; Template DNA, 10 ng; Supplement ddH2O to 20 μL. Reaction parameters: Pre-denaturation at 95 °C (3 min); Denaturation at 95 °C (30 s), annealing at 52 °C (30 s), elongation at 72 °C (45 s), 27 cycles; Extension at 72 °C (10 min). After amplification, the PCR products were subjected to 2% agarose gel electrophoresis, underwent quantitative fluorescence analysis, and were finally mixed in accordance with the sequencing volume requirements. The library was constructed with the TruSeqTM DNA Sample Prep Kit (Illumina, San Diego, CA, USA) and sequenced on the MiSeq PE300 platform (Illumina, San Diego, CA, USA). The data acquired from MiSeq sequencing consisted of paired-end sequences, which were spliced into a single sequence using FLASH 1.2.11 software. The quality of the reads and the effect of splicing were quality-controlled using Fastp 0.19.6 software. Concurrently, Fastp 0.19.6 software was also used to assess the quality of the reads and the splicing effect. Samples were differentiated based on the barcodes at the beginning and end of the sequences and the primer sequences to obtain the valid sequences for each sample.

### 2.5. Data Processing and Analysis

ANOVA was conducted using SPSS 27.0 software to evaluate the indicators of physical and chemical properties, enzyme activity, and bacterial Alpha diversity index of surface soil across various measurements and bare land. The Duncan test was employed to identify significant differences, while a t-test was used to analyze the significant differences in the above-mentioned indicators at different soil depths. Uparse 7.0.1090 software was used to perform OTU clustering of the effective sequences based on 97% similarity. Mothse 7.0.1090 software was used to perform OTU clustering of the effective sequences. Mothur 1.30.2 software was utilized to calculate the bacterial community Alpha diversity indices of all samples. R language 3.3.1 software was employed to test the difference between groups of Chao, Ace, Shannon, Simpson, and Coverage indices and construct the PCoA analysis plots, the soil bacterial community composition plots, correlation heatmaps, and RDA analysis plots. Kruskal–Wallis rank-sum tests were used to analyze the differences in relative abundance of major bacterial phyla among samples. PICRUSt2 2.2.0 software, based on the Kyoto Encyclopedia of Genes and Genomes (KEGG) database, was used for functional prediction of sample bacterial communities. Post hoc testing methods were used to conduct pairwise significant difference analysis between the relative abundance of primary and secondary functions of soil bacterial communities in the four afforestation treatment areas and bare land.

## 3. Results

### 3.1. Analysis of Soil Particle Composition

**Figure 2:**
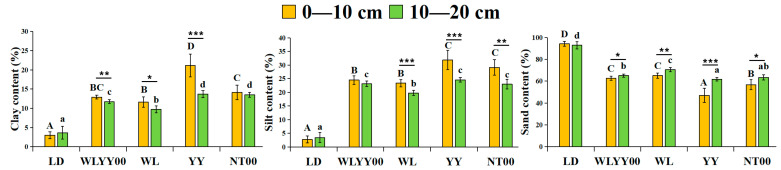
The differences in the particle composition characteristics of surface soil between different regions. Different uppercase letters indicate significant differences in the 0–10 cm soil layer for the same index between different points, while different lowercase letters indicate significant differences in the 10–20 cm soil layer for the same index between different points. * 0.01 < *p* ≤ 0.05, ** 0.001 < *p* ≤ 0.01, *** *p* ≤ 0.001. WLYY00: *Salix cheilophila* + *Populus simonii* plantation; WL: *S. cheilophila* plantation; YY: *P. simonii* plantation; NT00: *Caragana korshinskii* plantation; LD: bare land of flowing dune.

In the study area, the particle composition of the four afforestation measure areas and bare land surface soil was primarily dominated by sand particles, accounting for more than 47% (Figure 2). Among them, the sand content of the surface soil in the LD was the highest, exceeding 92%, while the sand content of the surface soil in the four afforestation practice areas was significantly smaller than that in LD, especially in the YY sample site, where the sand content of the soil layer at two depths was the lowest among all sample sites. Meanwhile, the contents of fine particles (sticky and powdery) in the surface soil of the four afforestation measures were significantly higher than those of the LD, while the YY sample plot had the highest fine particle content in the surface soil among all sample plots. This indicated that the YY measure had the optimal effect on the refinement of surface soil particles in sandy areas. The fine particle content of the 0–10 cm soil layer was higher than that of the 10–20 cm soil layer in the four afforestation measures areas. This characteristic indicated that the afforestation measures had a superficial tendency to refine soil particles, which better promoted the enrichment of fine particulate matter in the 0–10 cm soil layer.

### 3.2. Characteristics of Soil Physicochemical Properties

**Figure 3:**
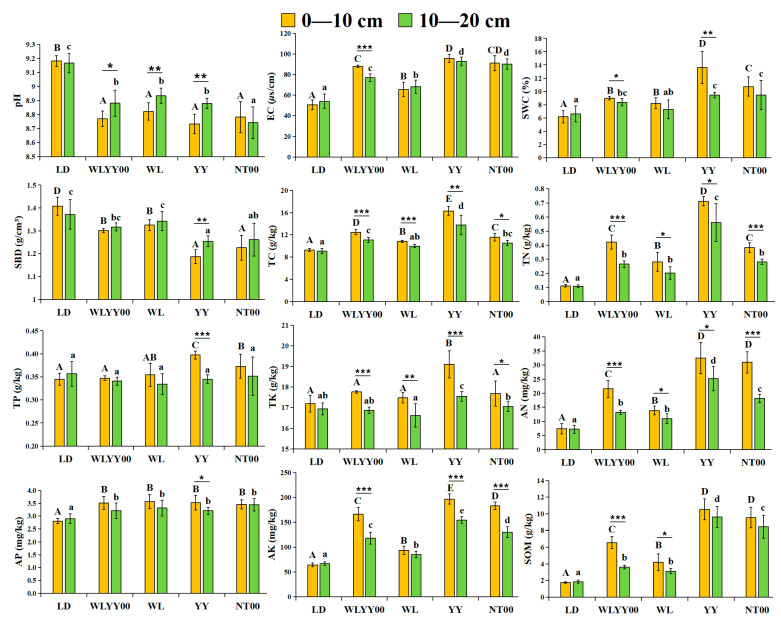
The differences in the physicochemical properties of surface soil at different locations. EC: electrical conductivity; SBD: soil bulk density; SWC: soil water content; TC: total carbon; TN: total nitrogen; SOM: soil organic matter; TP: total phosphorus; TK: total potassium; AN: alkali-hydrolyzable nitrogen; AP: available phosphorus; AK: available potassium. Different uppercase letters indicate significant differences in the 0–10 cm soil layer for the same index between different points, while different lowercase letters indicate significant differences in the 10–20 cm soil layer for the same index between different points. * 0.01 < *p* ≤ 0.05, ** 0.001 < *p* ≤ 0.01, *** *p* ≤ 0.001.

As observed from Figure 3, all four afforestation measures exerted significant improvement effects on the surface soil quality of the sandy land; however, evident differences were observed in their amelioration effects. The surface soil of each sample site was alkaline, with pH values exceeding 8.73. Compared with the bare land, the pH values of the surface soil in the four afforestation measure areas were significantly lower, while the EC values were significantly higher. Among them, the SWC of the surface soil in the WLYY00, YY, and NT00 sample plots was significantly higher than that of bare land, while the SBD was significantly lower than that of bare land. In the WL sample plot, the SWC of the 0–10 cm soil layer was significantly higher than that of bare land, while the SBD was significantly lower than that of bare land. Conversely, the SWC and SBD of the 10–20 cm soil layer were not significantly different from those of bare ground. Among the four afforestation measures, pH and SBD were the lowest, while the EC and SWC were the highest in the topsoil layer of the YY measure area. Longitudinal comparison of different soil layers revealed that the SWC of the 0–10 cm soil layer was higher than that of the 10–20 cm soil layer, while the SBD was lower than that of the 10–20 cm soil layer at each sample site. In the WLYY00, WL, and YY sample plots, the pH of the 0–10 cm soil layer was significantly lower than that of the 10–20 cm soil layer, while the EC was significantly higher in the 0–10 cm soil layer than that in the 10–20 cm soil layer at the WLYY00 sample site.

The LD, TN, AN, AP, AK, and SOM contents of the surface soil in the four afforestation measure areas were significantly higher, while the WLYY00, YY, and NT00 measures significantly elevated the TC contents of the surface soil. The WL measure only significantly increased the TC contents of the 0–10 cm soil layer. In addition, YY and NT00 significantly increased the TP content in the 0–10 cm soil layer, while the YY measure also significantly enhanced the TK content. Among the four afforestation measures, the TC, TN, TP, TK, AN, AP, AK, and SOM contents of the surface soil in the YY sample plots were the highest, thereby indicating that the YY measure was more effective in enhancing soil nutrient contents. Longitudinal comparison of different soil layers revealed that the nutrient content of the 0–10 cm soil layer was generally higher than that of the 10–20 cm soil layer, while the soil nutrients showed an evident aggregated surface effect.

### 3.3. Characteristics of Soil Enzyme Activities

**Figure 4:**
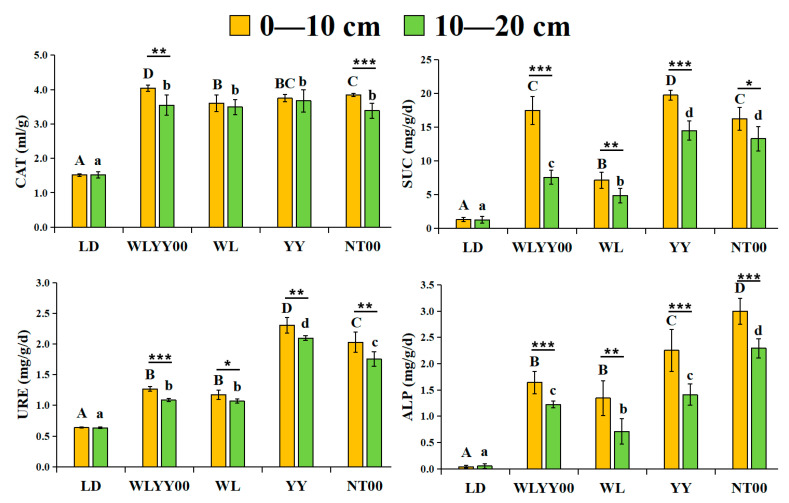
The differences in enzyme activity characteristics of surface soil between different regions. CAT: Catalase; SUC: Sucrase; URE: Urease; ALP: Alkaline phosphatase. Different uppercase letters indicate significant differences in the 0–10 cm soil layer for the same index between different points, while different lowercase letters indicate significant differences in the 10–20 cm soil layer for the same index between different points. * 0.01 < *p* ≤ 0.05, ** 0.001 < *p* ≤ 0.01, *** *p* ≤ 0.001.

As observed from Figure 4, the CAT, SUC, URE, and ALP activities in the surface soil of the four afforestation measures were significantly higher than those in the bare land. In the distribution of vertical soil layers, the activities of these four enzymes in the 0–10 cm soil layer were higher than those in the 10–20 cm soil layer, thereby indicating an evident clustering effect. Different afforestation measures exhibited their own advantages in the enhancement effect of surface soil enzyme activities, with WLYY00 measures improving surface soil CAT activity more significantly, followed by NT00 and YY measures. SUC and URE activities were most prominently enhanced by YY measures, followed by NT00 measures. ALP activity was most significantly enhanced by NT00 measures, followed by YY measures.

### 3.4. Correlation Analysis of Physical and Chemical Properties and Enzyme Activities

**Figure 5:**
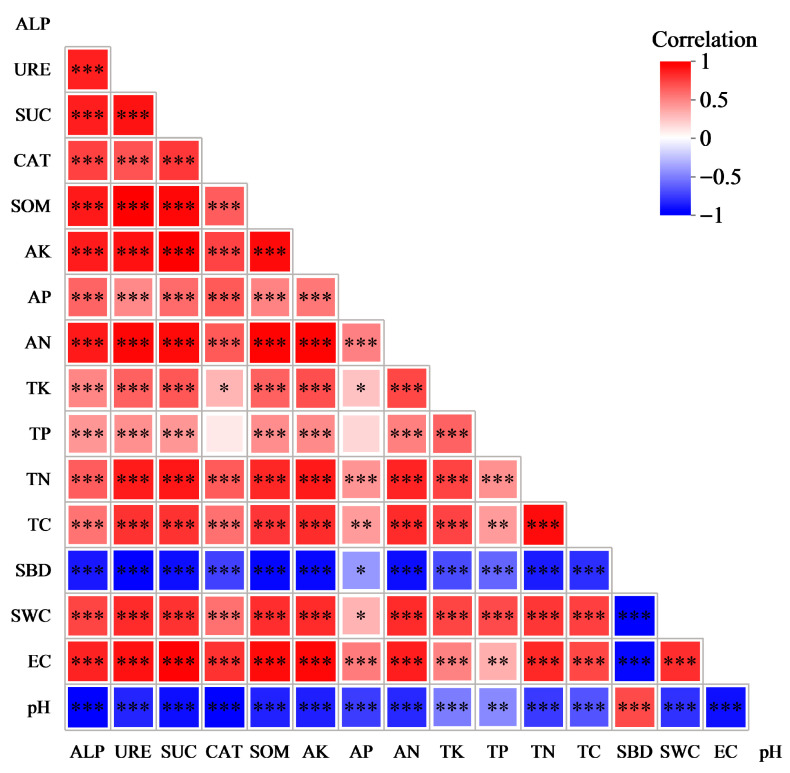
Correlation between physical and chemical properties and enzyme activities of soil samples. In the heatmap, red represents positive correlation, while blue denotes negative correlation. The color intensity indicates the magnitude of positive or negative correlation, while the asterisks in color blocks refer to significance levels. * 0.01 < *p* ≤ 0.05, ** 0.001 < *p* ≤ 0.01, *** *p* ≤ 0.001.

As observed from Figure 5, significant correlations existed between most of the indicators of physicochemical properties and enzyme activities in the surface soil of sandy land. sBD and pH were significantly positively correlated, while they were significantly negatively correlated with the rest of the indicators of physicochemical properties and enzyme activities. TP, CAT, and AP had no significant correlation; however, they were significantly positively correlated with the other indicators of physicochemical properties and enzyme activities. Except for SBD, pH, and TP, all the other physicochemical indicators and enzyme activities exhibited significant positive correlations with each other.

### 3.5. Characterization of Soil Bacterial Community Structure

#### 3.5.1. Statistics of OTUs

**Figure 6:**
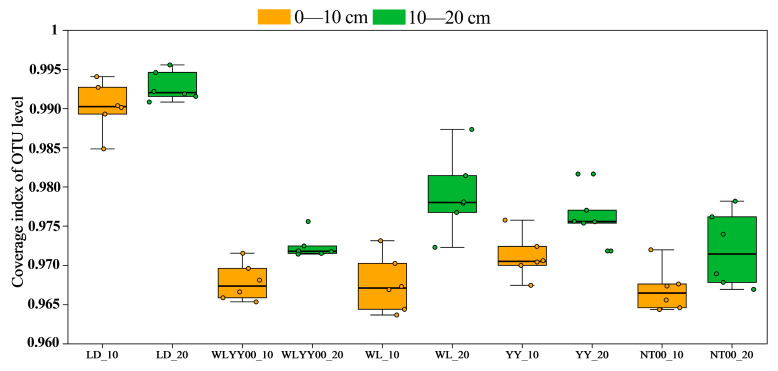
Bacterial community coverage indices of soil samples based on OTU level.

A total of 2,718,120 valid sequences were obtained from 60 soil samples after bacterial sequencing and quality control splicing. The sequences were clustered, transformed, compared with the related species database, and annotated to obtain 20,108 OTUs. These belonged to 53 phyla, 158 classes, 401 orders, 672 families, and 1405 genera. At the OTU level, the bacterial community coverage index of each soil sample was above 0.96 (Figure 6), indicating that the current sequencing volume could adequately cover most of the species of the bacterial community in the surface layer of the four afforestation measures and comprehensively reflect their species composition characteristics.

#### 3.5.2. Alpha Diversity Index

**Figure 7:**
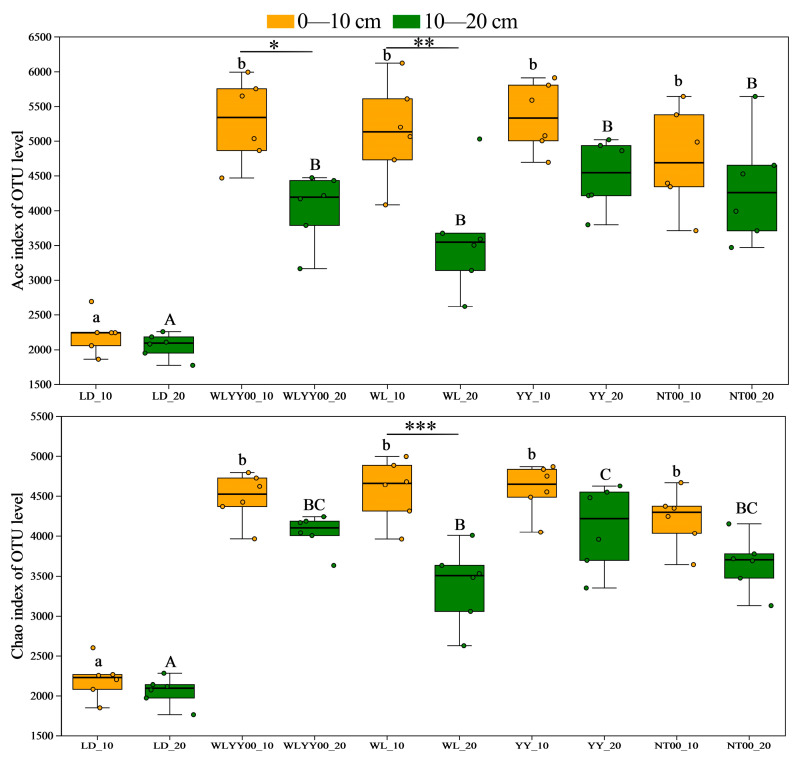
The differences in the richness index of bacterial communities in the surface soil between different regions based on OTU level. Different uppercase letters indicate significant differences in the 0–10 cm soil layer for the same index between different points, while different lowercase letters indicate significant differences in the 10–20 cm soil layer for the same index between different points. * 0.01 < *p* ≤ 0.05, ** 0.001 < *p* ≤ 0.01, *** *p* ≤ 0.001.

**Figure 8:**
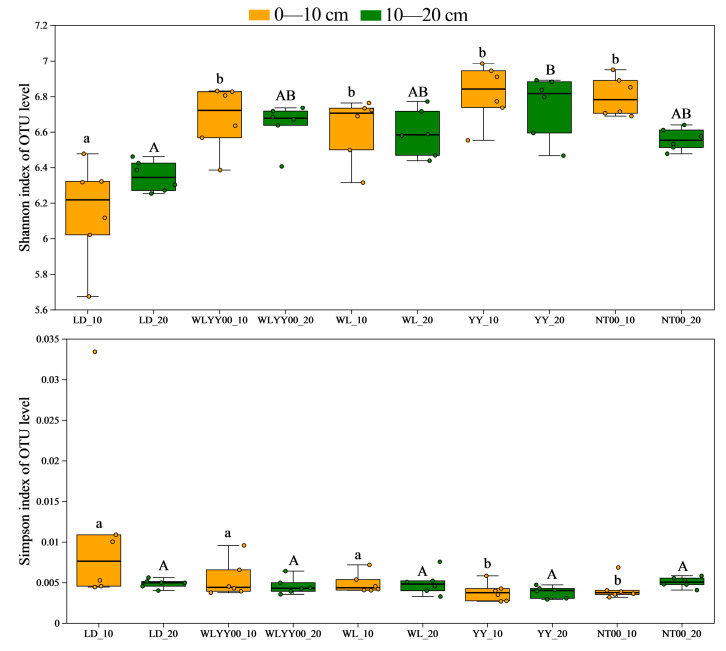
The differences in the diversity index of bacterial communities in the surface soil between different regions based on OTU level. Different uppercase letters indicate significant differences in the 0–10 cm soil layer for the same index between different points, while different lowercase letters indicate significant differences in the 10–20 cm soil layer for the same index between different points.

Alpha diversity analysis primarily evaluated species richness and community diversity characteristics of soil bacterial communities at various sites through several sets of core indices. Among them, the Ace and Chao indices were the key indices used to quantify the species richness of the community (Figure 7; higher index values represent higher bacterial community richness). The Shannon and Simpson indices were used to characterize the level of diversity of the community (Figure 8); the higher the Shannon index and the lower the Simpson index, the richer the diversity of the community. As observed from Figure 7 and Figure 8, the Ace, Chao, and Shannon indices of the surface soil bacterial communities in the four afforestation measures were significantly higher than those of the LD, while the Simpson indices were lower than those of the LD. The Ace, Chao, and Shannon indices of the 0–10 cm soil layer were higher than those of the 10–20 cm soil layer, while the Simpson indices were lower than those of the 10–20 cm soil layer. This indicates that the four afforestation measures can significantly increase the abundance and diversity of bacterial communities in the surface layer of alpine sandy soils, while the distribution characteristics of bacterial species abundance and diversity among different soil layers were similar to changes in their physicochemical properties and enzyme activities. This also indicated that the abundance and diversity of the 0–10 cm soil layer were greater than that of the 10–20 cm soil layer. Among the four afforestation measures, the Ace, Chao, and Shannon indices were the highest, while the Simpson index was the lowest in the YY sample site. This indicated that the YY measure exerted the most prominent effect on enhancing the abundance and optimizing the diversity of the surface soil bacterial community in alpine sandy land.

#### 3.5.3. Soil Bacterial Community Composition

**Figure 9:**
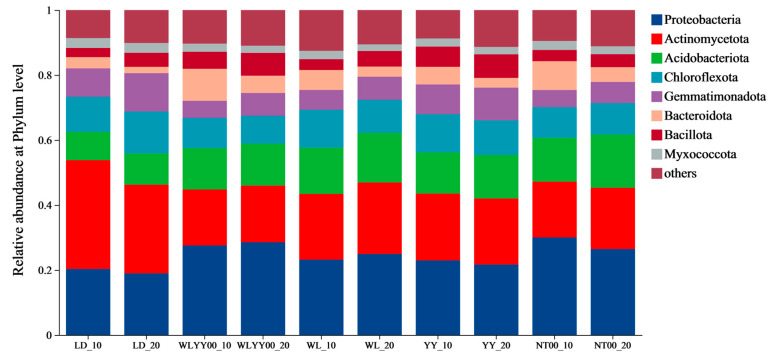
The differences in the composition of bacterial communities in the surface soil between different regions at the phylum level. Others represent the combined bacterial phyla ranked beyond 8th in relative abundance from high to low.

The surface soil bacterial communities in the four afforestation measure areas and bare land mainly contained eight bacterial phyla (Figure 9), with relative abundances above 2%. Among these phyla, Proteobacteria, Actinomycetota, Acidobacteriota, and Chloroflexota were the dominant phyla, with relative abundances of 19.0–30.0%, 17.2–33.5%, 8.7–16.4%, and 8.6–12.9%, respectively. The relative abundance of Actinomycetota was lower than that of the LD, while the relative abundance of Proteobacteria was higher than that of the LD in the four afforestation measure areas at the same soil depth, especially in the WLYY00 and NT00 sample plots. In these plots, the relative abundance of Proteobacteria in the surface layer of the soil exceeded 26.4%. The relative abundance of Chloroflexota, Bacteroidota, and Myxococcota in the 0–10 cm soil layer was higher than that in the 10–20 cm soil layer in the four afforestation measure areas. Specifically, the relative abundance of Bacteroidota exhibited a larger difference between the two soil layers. Acidobacteriota, Gemmatimonadota, and Bacillota all had relative abundances lower than the 10–20 cm soil horizon; however, the difference in relative abundance between the two soil horizons was smaller for these three bacterial phyla.

#### 3.5.4. PCoA Analysis

**Figure 10:**
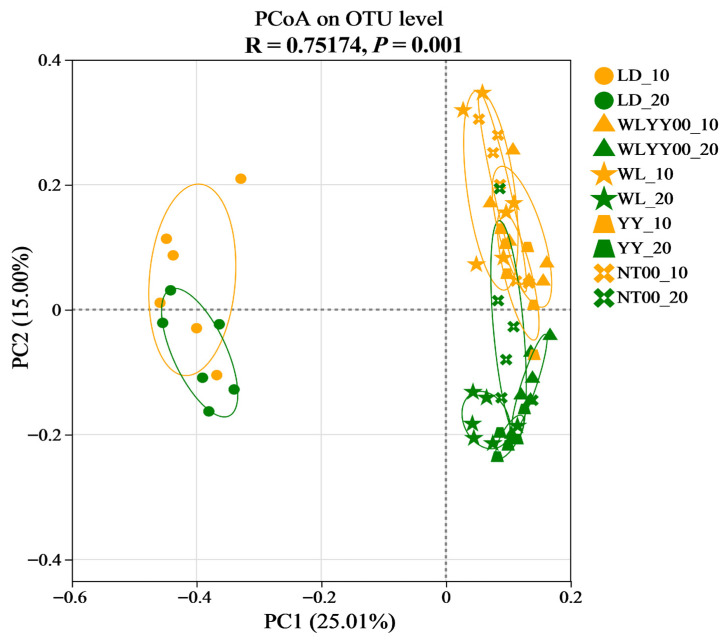
Graph of PCoA analysis based on the OTU level.

The PCoA plot visualizes the similarity of bacterial community composition among different samples; the closer the distance between sample points, the higher the degree of similarity. As shown in Figure 10 (R = 0.75174, *p* = 0.001), the samples of two soil depths in the LD were closer to each other, while both samples were farther away from the surface soil samples of the four afforestation practice areas. This distribution characteristic indicated that the bacterial community composition of the two soil layers in the LD was similar, while the bacterial community of the surface soil in each afforestation measure area was different. The distribution of the surface soil samples in the four afforestation measure areas was more aggregated, indicating that the bacterial community composition of the surface soil across different afforestation measure areas was less different and showed higher similarity overall. Further observation showed that the sample distribution in the same soil layer was more aggregated among the four afforestation measure areas, thereby indicating that the soil bacterial community compositions across different afforestation practice areas were more consistent within the same soil depth. Together, these results indicated that the implementation of afforestation measures could significantly change the composition of surface soil bacterial communities on alpine sands; however, the difference in the type of afforestation measures exerted a relatively minor effect on their composition.

#### 3.5.5. Significance Test of Differences Between Major Bacterial Phyla Groups

**Figure 11:**
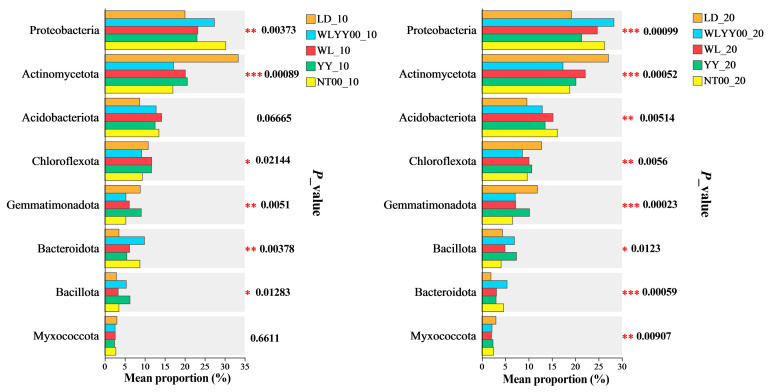
Test of differences among major bacterial phyla. * 0.01 < *p* ≤ 0.05, ** 0.001 < *p* ≤ 0.01, *** *p* ≤ 0.001.

The relative abundances of the major bacterial phyla in the surface soil bacterial communities of the four afforestation measures and bare land were tested for the significance of intergroup differences (Figure 11). The results showed that the relative abundance of most of the major bacterial phyla differed significantly among the various afforestation measures and bare land. Moreover, the relative abundances of Acidobacteriota and Myxococcota did not show any significant differences in the 0–10 cm soil layer at the sampled sites. These results indicated that the type of afforestation measures significantly regulated the relative abundance of most of the major bacterial phyla in the surface soil. Combined with the results of PCoA analysis, it can be concluded that, in the alpine sandy land, different afforestation measures exerted a minor effect on the overall composition of the bacterial community in the surface soil; however, they had a significant effect on the relative abundance of the major bacterial phyla in the surface soil.

### 3.6. Correlation Analysis of Physical and Chemical Properties, Enzyme Activities, and Bacterial Community Structure

**Figure 12:**
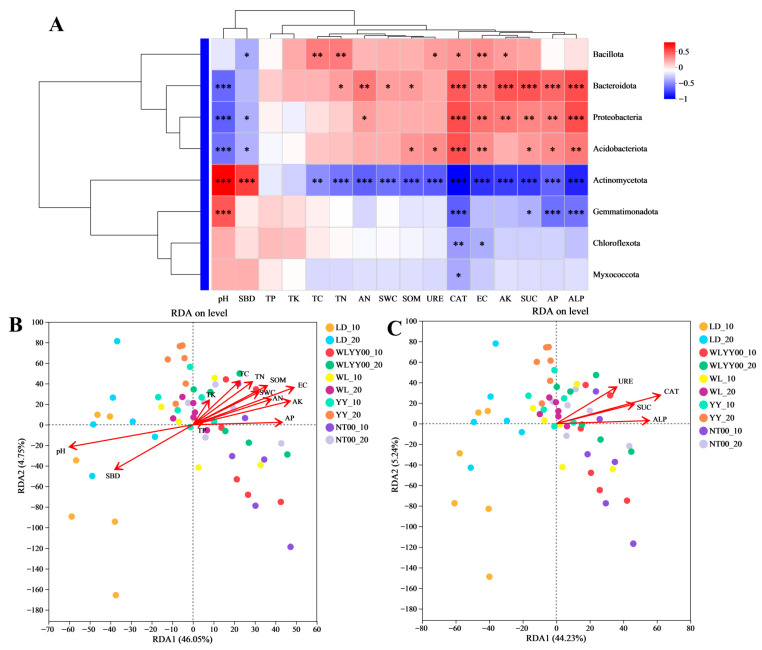
Correlation between physicochemical properties and enzyme activity indices and bacterial community structure. (**A**) shows the heat map of the correlation between physical and chemical properties and enzyme activities and the relative abundances of major bacterial phyla; (**B**) shows the RDA analysis of physical and chemical properties and bacterial community structure; (**C**) shows the RDA of enzyme activities and bacterial community structure. * 0.01 < *p* ≤ 0.05, ** 0.001 < *p* ≤ 0.01, *** *p* ≤ 0.001.

Correlation analysis was performed between the physical and chemical properties and enzyme activity indices of the surface soil and its bacterial community structure in the research sample plots (Figure 12). The results showed that most physical and chemical properties and enzyme activity indices had a significant effect on the bacterial community structure. The relative abundance of Bacillota was significantly positively correlated with AK, EC, CAT, URE, TN, and TC and negatively correlated with SBD, as evidenced by Figure 12A. The relative abundances of Bacteroidota, Proteobacteria, and Acidobacteriota were significantly negatively correlated with ALP, AP, SUC, EC, and CAT, and negatively correlated with pH. Among these, the relative abundances of Bacteroidota and Proteobacteria were also significantly positively correlated with AK and AN, while the relative abundances of Bacteroidota and Acidobacteriota were additionally significantly positively correlated with SOM. The relative abundance of Bacteroidota was significantly positively correlated with TN and SWC, while the relative abundances of both Proteobacteria and Acidobacteriota were significantly negatively correlated with SBD. The relative abundance of Acidobacteriota was significantly positively correlated with URE. The relative abundance of Actinomycetota was significantly positively correlated with pH and SBD, not significantly correlated with TP and TK, and significantly negatively correlated with all remaining indicators. The relative abundance of Gemmatimonadota was significantly negatively correlated with CAT, SUC, AP, and ALP and positively correlated with pH. The relative abundances of Chloroflexota and Myxococcota were significantly negatively correlated with CAT, while the relative abundance of Chloroflexota was also negatively correlated with EC. As shown in Figure 12B (RDA1 46.1%, RDA2 4.8%), pH was the most critical physicochemical factor affecting the structure of the surface soil bacterial community in the four afforestation measures areas and bare land with a restoration period of 24 years, followed by EC. As shown in Figure 12C (RDA1 44.2%, RDA2 5.2%), the four enzyme activities had a significant effect on the structure of the surface soil bacterial community of the study sample sites, with CAT having the most prominent effect.

### 3.7. Functional Prediction of Surface Soil Bacterial Community

#### 3.7.1. Primary Function Prediction

**Figure 13:**
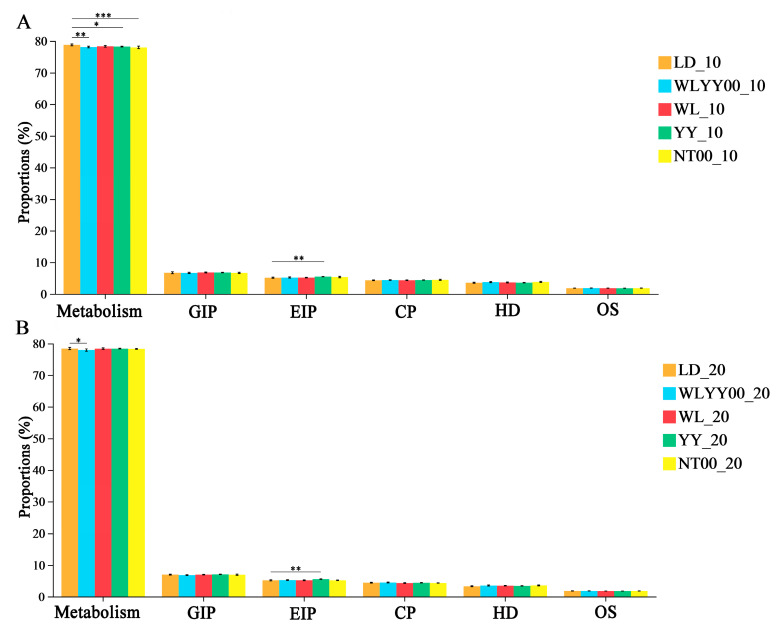
Relative abundances of the primary functional pathways in soil bacterial communities. (**A**) shows the relative abundances of bacterial communities in the 0–10 cm soil layer; (**B**) shows the relative abundances of bacterial communities in the 10–20 cm soil layer; Significance markers are the results of the post hoc test for two-by-two comparisons between the sample plots and the bare ground in each area. GIP: Genetic Information Processing; EIP: Environmental Information Processing; CP: Cellular Processes; HD: Human Diseases; OS: Organismal Systems. * 0.01 < *p* ≤ 0.05, ** 0.001 < *p* ≤ 0.01, *** *p* ≤ 0.001.

The surface soil bacterial community across various afforestation measures and bare land exhibited six functions at the primary function prediction level. The relative abundance of each function was highly redundant, with values greater than 1%. Notably, the metabolism function had the highest relative abundance of more than 77% (Figure 13). The results of the post hoc test showed that, compared with the LD, the WLYY00 measure significantly reduced the surface soil bacterial community. Moreover, the WLYY00 measure significantly reduced the relative abundance of metabolism in the surface soil bacterial community, while the YY and NT00 measures only significantly reduced the relative abundance of metabolism in the 0–10 cm soil layer. The relative abundance of metabolism in the surface soil in the WL measure area was not significantly different from that in the LD. In addition, the relative abundances of environmental information processing in the surface soil in the YY measure area were all significantly higher than those in the LD, thereby indicating that the YY measure assisted the bacterial community in the surface soil of the sandy land to fulfill the function of environmental information processing as a primary function.

#### 3.7.2. Secondary Function Prediction

**Figure 14:**
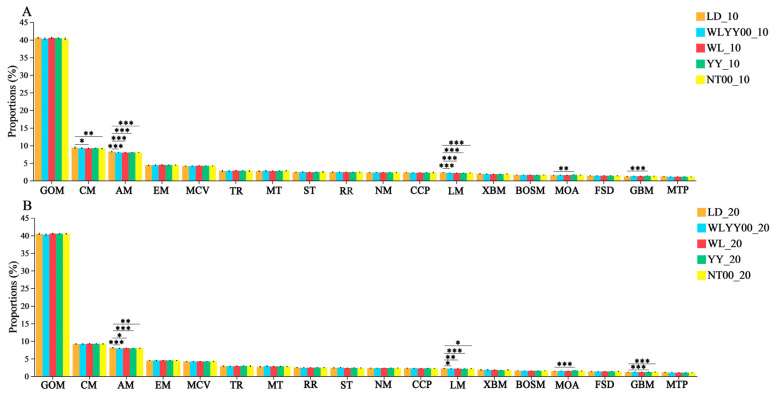
Relative abundance of secondary functional pathways in soil bacterial communities. (**A**) shows the relative abundances of the main secondary functions of the bacterial community in the 0–10 cm soil layer; (**B**) shows the relative abundances of the main secondary functions of the bacterial community in the 10–20 cm soil layer; Significance markers are the results of the two-by-two post hoc test. GOM: global and overview maps CM: Carbohydrate metabolism; AM: Amino acid metabolism; EM: Energy metabolism; MCV: Metabolism of cofactors and vitamins; TR: Translation; MT: Membrane transport; RR: Replication and repair; NM: Nucleotide metabolism; CCP: Cellular community—prokaryotes; ST: Signal transduction; LM: Lipid metabolism; XBM: Xenobiotics biodegradation and metabolism; BOSM: Biosynthesis of other secondary metabolites; MOA: Metabolism of other amino acids; FSD: Folding, sorting and degradation; GBM: Glycan biosynthesis and metabolism; MTP: Metabolism of terpenoids and polyketides. * 0.01 < *p* ≤ 0.05, ** 0.001 < *p* ≤ 0.01, *** *p* ≤ 0.001.

Among the primary functions of soil bacterial community distributed in different afforestation treatment areas and bare ground surface layer, 18 secondary functions were observed with relative abundances greater than 1% (Figure 14). The relative abundance of each function was also characterized by a high degree of redundancy, while the main secondary functions were global and overview maps (39.9–40.7%), carbohydrate metabolism (8.9–9.7%), amino acid metabolism (7.9–8.4%), and energy metabolism (4.3–4.6%). The results of the post hoc test showed that amino acid metabolism and lipid metabolism of the surface soils in all the measurement areas were highly redundant. Metabolism and lipid metabolism were significantly lower than those of the LD, while the relative abundances of carbohydrate metabolism in the 0–10 cm soil layer of the WL and NT00 measure areas were also significantly lower than those in the LD. This indicated that the construction of afforestation measures would significantly inhibit the bacterial community in the topsoil layer of the sandy land. Amino acid metabolism and lipid metabolism in the WL and NT00 measure areas also significantly inhibited the carbohydrate metabolism function of the bacterial community in the 0–10 cm soil layer. In addition, the relative abundances of metabolism of other amino acids and glycan biosynthesis and metabolism were significantly higher in the surface soil of the YY measure area than those in the LD. This indicated that the YY measure could be beneficial for promoting the metabolism of other amino acids and glycan biosynthesis and metabolism of the bacterial community of the surface soil to fulfill the secondary function of metabolism of other amino acids and glycan biosynthesis.

## 4. Discussion

### 4.1. Differentiation Characteristics of Surface Soil Particle Composition Across Distinct Afforestation Treatment Areas

Soil serves as the substrate for plant growth and development, and it essentially consists of a diverse range of particle sizes. These soil particles influence key properties, such as permeability, nutrient retention, and water-holding capacity by affecting air-circulation efficiency, nutrient transformation processes, and hydrophobic characteristics [31,32]. In wind-sand environments, long-term wind erosion reduces the fine particle content of sandy surface soils and increases the proportion of sand particles in the particle composition, resulting in a decline in soil quality [33]. The surface soil particle composition across all samples was dominated by sand, indicating that on the alpine sands of the Gonghe Basin, the fine particulate matter of surface soils was susceptible to wind transport and loss and more severely affected by wind erosion. Compared with the LD, the sand content of the four afforestation measures decreased significantly, while the content of fine particles (sticky particles + powder particles) increased significantly. This was consistent with the findings of Li et al. [34], who also found that afforestation could influence soil particle composition toward finer particles in sandy areas, as evidenced by their analysis of the effects of various establishment years of afforestation measures on the surface soil particle composition in the Tengger Desert. The vegetation established through afforestation measures mitigates the kinetic energy of wind and the sand-carrying capacity of wind-sand flow. Additionally, the presence of an apoptotic cover on the soil surface further isolates the wind from directly scouring the surface soil. This process effectively reduces wind erosion on the surface soil in sandy areas, minimizes the loss of fine particulate matter, and enhances the current situation of coarse-grained soil granularity. These observations underscore the substantial positive effects of afforestation measures in combating wind erosion [35,36,37]. Among the four afforestation measures, the surface soil of the YY area exhibited the highest fine particle content. This indicated that the measure effectively enhanced wind erosion resistance in sandy soil and promoted the development of soil fine granularity, thereby creating more favorable soil conditions for subsequent improvements in soil fertility and vegetation growth in the area. The study revealed that the fine particle content in the 0–10 cm soil layer was higher than that in the 10–20 cm layer, while the sand particle content was significantly lower than that in the 10–20 cm layer in all the sampled areas. Research by Li et al. [38] in the Kubuqi Desert indicated a gradual increase in the fine particle content with increasing soil depth. The fine particle content gradually decreased with increasing soil depth. Therefore, the four types of afforestation measures with a restoration period of 24 years could significantly enhance the particle composition of the surface soil in the sandy area, particularly in the 0–10 cm layer, with the YY measure demonstrating the most effective results.

### 4.2. Characteristics of the Physicochemical Properties and Enzyme Activities of Surface Soils Across Various Afforestation Practices

Soil physicochemical properties quantitatively characterize the ecological roles played by the soil. Parameters, including pH, EC, SBD, and SWC, influence the alkalinity, salt accumulation, aeration, and water retention of sandy soils [39]. Additionally, SOM and nitrogen, phosphorus, and potassium contents are the core components of soil nutrient content [40]. Soil enzymes influence soil fertility formation, promote soil biochemical reactions, and drive soil nutrient cycling and energy conversion. These processes directly influence the efficiency of effective nutrient supply to the soil, subsequently affecting nutrient uptake and utilization by vegetation [41]. Therefore, soil physical and chemical properties and enzyme activity indicators constitute core indicators for assessing soil quality. The surface soils of all sites in this study were alkaline. The pH was similar to that of surface soils in the Tengger Desert [42]. Compared with the LD, the pH of the surface soil was significantly reduced in all areas, with the pH of the 0–10 cm soil layer significantly lower than that of the 10–20 cm soil layer in the WLYY00, WL, and YY sample plots. Conversely, the pH of the two soil layers did not show a significant difference in the NT00 sample plot. This suggests that afforestation measures can effectively slow down the rate of alkalization in sandy soils, with a more pronounced effect observed in the 0–10 cm soil layer across most of the areas (WLYY00, WL, and YY). The EC of the surface soils in the four afforestation measure areas was significantly higher than that in the LD. This is also similar to the findings of Chang et al. [43] in the Tengger Desert, attributed to the presence of abundant apoptotic material on the ground surface of the LD. Following the decomposition of the apoptotic material by microorganisms, the nutrient ions carried by the apoptotic material are integrated into the soil, leading to increased EC values [44]. The increase in the EC value of sandy soil negatively affects the colonization and propagation of non-saline herbaceous plants, highlighting the potential limitations of afforestation measures in improving the sandy environment. In terms of SBD and SWC, a smaller SBD corresponds to a higher SWC This may be attributed to soils with smaller SBDs exhibiting higher porosity, enabling them to adsorb and store more water, thereby leading to higher SWC [45]. The correlation heat map of the physicochemical properties and enzyme activities of this study also showed a significant negative correlation between SBD and SWC. Compared with the LD, the SBD of the surface soil in all areas significantly decreased, while the SWC significantly increased. This indicated that the afforestation measures improved the physical environment of the surface soil in the sandy area by introducing vegetation. The root system of vegetation continuously compresses and fractures the soil throughout the growth process, thereby enhancing aeration and water retention. Additionally, withered vegetation creates a mulch layer on the soil surface, which mitigates the impact of raindrops, prevents compaction and clogging of pore spaces, and reduces water evaporation. The surface soil in the YY measure area exhibited the highest SWC and the lowest SBD, indicating that the YY measure had the most prominent impact on enhancing the physical properties of sandy soil among all afforestation measures. In addition, the SWC of the 0–10 cm soil layer was higher than that of the 10–20 cm soil layer, while the SBD of the 0–10 cm soil layer was lower than that of the 10–20 cm soil layer. This indicated that the afforestation measures positively affected the physical properties of the 0–10 cm soil layer.

The four afforestation measures significantly affected the improvement of soil nutrients and enzyme activities in sandy soils, aligning with the findings of Li et al. [46] in the sandy areas of Horqin. Compared with the LD, most soil nutrient contents and soil enzyme activities were significantly elevated following 24 years of restoration under the four afforestation measures. Further analysis revealed that the YY measure was the most effective in improving both soil nutrient contents and enzyme activities, and it also increased the TK content of the sandy surface soil independently. This may be attributed to the sand-fixing vegetation in the YY measure comprising *P. simonii*. Over a growth cycle of 24 years, the *P. simonii*, as a tree, exhibits a greater density of root lengths compared with the sand-fixing plants in other measures. This characteristic allows for denser interspersion with soil pores to enhance the stability of the soil structure. Additionally, the larger root system contact area facilitates the secretion of organic acids and substances, which further improve activation efficiency and microbial colonization, thereby enhancing soil nutrient content and enzyme activity. The secretion of additional organic acids and substances via a larger root contact area further enhances soil nutrient activation efficiency and microbial colonization, thereby strengthening support for soil nutrient accumulation and enzyme activity [47]. In terms of soil physical and chemical properties and enzyme activities, the YY measure demonstrated a significantly greater overall improvement effect on sandy soil over a restoration period of 24 years compared to that observed for the other three measures, indicating a more optimal vegetation allocation at this restoration stage. The nutrient content and enzyme activity of the 0–10 cm soil layer were higher than those of the 10–20 cm soil layer, indicating a pronounced aggregated surface effect. This finding aligns with the soil nutrient characteristics observed at varying depths of the soil layers in the *Populus euphraticu* woodland in the oasis–desert transition zone at the edge of the Taklamakan Desert [48]. In arid desert ecosystems, the root systems of afforestation plants absorb nutrients from sandy soil. These nutrients return to the surface layer through apoptosis. Following decomposition by microorganisms, the limited water availability and low fertilizer retention capacity of the soil hinder nutrient migration to deeper layers. Consequently, nutrients are mainly retained in the surface layer, leading to surface aggregation effects.

### 4.3. Differentiation Characteristics of Surface Soil Bacterial Community Structure and Function Across Various Afforestation Measures

The microbial community is an important component of soil, facilitating material exchange and energy transfer between various layers [49]. Among them, bacteria are the most abundant, numerous, and functional soil microorganisms, and exhibit the highest metabolic activity in sand ecosystems. They have a beneficial effect in enhancing soil nutrient content and enzyme activities [50]. Proteobacteria, Actinomycetota, Acidobacteriota, and Chloroflexota emerged as the dominant phyla in the four afforestation measures and bare land surface soil of this study with a restoration period of 24 years. This is similar to the dominant phyla in the surface soils of the sandy areas of Poyang Lake [51] and those of Horqin [52]. Although this study area was an alpine sandy area with high altitude and low temperature, the special habitat did not have a decisive influence on the composition of the dominant phyla in the surface soil of sandy ecosystems. This observation indicates that these dominant phyla exhibit considerable adaptability to sandy habitats across varying altitudes and temperature gradients, serving as the core taxa maintaining the structure and function of soil microbial communities in sandy soils. Compared with the LD, the four afforestation measures significantly increased the abundance of surface soil in the sand areas. Furthermore, the bacterial community abundance in the 0–10 cm soil layer was higher than that in the 10–20 cm soil layer, aligning with the findings of An et al. [53] in the transition zone of the Northwest Desert Oasis and Tian et al. [54] in the Maowusu Sandy Land. This may be attributed to the higher nutrient content and enzyme activity in the topsoil layer of the afforestation measure area, especially in the 0–10 cm soil layer. This creates a more suitable environment for bacterial survival, thereby significantly enhancing the abundance of the soil bacterial community. The analysis of intergroup differences among major bacterial phyla revealed significant variations in their relative abundance across surface soils from different sites. This indicated that the regulatory effect of afforestation measures in enhancing the abundance of bacterial communities in the surface soil of sandy areas was primarily reflected in enhancing the relative abundance of the major bacterial phyla. Consequently, the overall enhancement of soil bacterial community abundance was realized by enhancing the abundance levels of major bacterial groups. In addition to increasing the abundance of bacterial communities, the four afforestation measures also significantly increased the diversity within the surface soil layer of the sandy land. Notably, the diversity of bacterial communities in the 0–10 cm soil layer was higher than that in the 10–20 cm soil layer, aligning with the characteristics of bacterial communities in *Caragana korshinskii* sand fixation forests and bare land in the Maowusu sandy land, as reported by Yang et al. [55]. The higher bacterial diversity in the surface soil layer of the afforestation measure area compared to that of the 10–20 cm soil layer supports this finding. This higher nutrient content and enzyme activity in the surface soil (especially in the 0–10 cm soil layer) contributed to this phenomenon in the afforestation measure area. In addition, the PCoA indicated a significant difference in bacterial community composition between the topsoil of the afforestation measure area and that of the LD. This difference was consistently observed in both the 0–10 cm and 10–20 cm soil layers. Furthermore, the bacterial community abundance and diversity of the topsoil of the YY area were higher than those of the other three sites, indicating that the YY measure was more effective in optimizing the habitat of the topsoil of sandy land. This indicates that the YY measure is more effective in optimizing the topsoil habitat and facilitating the development of bacterial communities in sandy areas. Soil bacterial function prediction indicated that the primary function of the surface soil across all sample sites was primary metabolism. The relative abundance of most secondary functions exhibited a high degree of redundancy, suggesting a conservative bacterial function in the surface soil of the study sample sites. Additionally, findings by Ye et al. [56] in the Tengger Desert revealed that the bacterial community function in sandy soils exhibited a conservative nature. At the first level of functional prediction, the metabolism function of the surface soil across the four afforestation measures was slightly weaker than that in the LD. Conversely, the environmental information processing function of the surface soil in the YY measure area was significantly stronger than that in the LD. This indicated that the nutrients in the surface soil in the afforestation measures area were high, allowing bacterial communities to operate without intensive metabolic activity to obtain resources, resulting in a slightly weaker metabolism function compared to that in the LD and a slightly more conservative bacterial community function. Consequently, the metabolism function was slightly weaker, whereas the YY measure could potentially strengthen the environmental information processing function by improving soil structure or micro-environmental regulation. This enhancement may facilitate the ability of the bacterial community to detect and respond to the environmental changes (e.g., nutrient dynamics and microclimate fluctuations), thereby further enhancing the environmental information processing function. At the level of secondary function prediction, the metabolism of other amino acids and glycan biosynthesis and metabolism functions in the surface soil of the YY area were significantly stronger than those of the LD. This suggests that this measure can optimize the material transformation capacity of the soil bacterial community in sandy soil and provide more robust microbial function support for soil nutrient cycling and biochemical processes. This also indicates that this measure can more precisely enhance the material transformation capacity of the soil bacterial community in sandy soils, thereby offering greater microbial function support for soil nutrient cycling and biochemical processes.

The structure of bacterial communities is closely linked to soil environmental factors, with changes in these factors having a substantial impact on the structural characteristics of bacterial communities [57]. In this study, pH was the primary environmental factor influencing the bacterial community structure in the surface soil of four afforestation measure areas with a restoration period of 24 years. Tian et al. [54] also concluded that pH was a key factor affecting the soil bacterial community structure in their study of the Maowusu sandy land. This suggests that pH plays a central regulatory role in soil bacterial community structure in sandy areas and indicates that pH may serve as a common environmental determinant influencing soil bacterial community structure in sandy areas, thereby exerting a generalized effect on screening and shaping bacterial communities across regions. In addition, all four enzyme activities had a strong influence on the bacterial community structure in the surface layer of sandy soil by accelerating the decomposition of soil organic matter and promoting nutrient transformation, which further contributed to the screening and structural regulation of the bacterial community [58]. However, there are still some other factors that were not directly explored in this study, such as other undetermined enzyme activities and trace element contents, which may also greatly affect the structure or composition of the soil bacterial community. Therefore, in the subsequent research, we need to supplement the determination of multiple soil factors to provide more comprehensive theoretical support for the technical optimization and ecological benefit assessment of sandy soil improvement.

## 5. Conclusions

(1) All four afforestation measures with a restoration period of 24 years significantly enhanced the particle composition, nutrient content, enzyme activity, and bacterial community structure (abundance and diversity) of the sandy surface soil. The improvement was particularly significant in the 0–10 cm soil layer, with the YY measure demonstrating the most effective results in sandy soil improvement.
(2) Functional prediction indicated that the primary function of the topsoil at each site was predominantly dominated by metabolism. The secondary functions were dominated by global and overview maps, with a high degree of redundancy observed across sites. Furthermore, the YY measure facilitated enhanced performance of topsoil in the primary functions of environmental and information processing and metabolism. Information processing was the primary function, and metabolism of other amino acids and glycan biosynthesis and metabolism were secondary functions.
(3) The pH was the primary environmental factor affecting the bacterial community structure of the topsoil in the area subjected to four afforestation measures over a restoration period of 24 years, with the four enzyme activities also exerting a more significant effect on its bacterial community structure.

## 6. Patents

[1] Shaobo Du; Huichun Xie; et al. A plant fixation device for desertification control in deserts. ZL202322873122.9, 26 July 2024.
[2] Shaobo Du; Huichun Xie; et al. An invention relates to a portable spraying device for desert algae biological control of sand. ZL202421424816.2, 28 March 2025.

## Figures and Tables

**Table 1:** Basic information of the sample site.

Sample Site	Longitude	Latitude	Elevation (m)	Area (m^2^)	Coverage
LD	100°14′28.266″ E	36°13′50.785” N	2822	273,333	nd
WLYY00	100°14′27.23″ E	36°15′32.432” N	2828	133,333	67%
YY	100°13′259.1″ E	36°15′32.713” N	2827	46,666	81%
WL	100°14′16.54″ E	36°14′50.678” N	2830	40,000	64%
NT00	100°14′36.26″ E	36°15′5.3167” N	2825	133,333	91%

Note: nd indicates no significance. WLYY00: *Salix cheilophila* + *Populus simonii* plantation; WL: *S. cheilophila* plantation; YY: *P. simonii* plantation; NT00: *Caragana korshinskii* plantation; LD: bare land of flowing dune.

**Table 2:** Soil physical and chemical properties, enzyme activity indexes, and their determination methods.

Index	Methods	Reference
Soil particle composition	Laser method (Mastersizer 2000 Laser Particle size analyzer, Malvern Company, Malvern, UK)	[23]
pH	Potentiometric method (water/soil ratio of 2.5:1, BPH-7100A pH meter, BELL Analytical Instruments Co., Ltd., Dalian, China)	[24]
Electrical conductivity (EC)	Conductometric method (water/soil ratio of 5:1 leaching, BEC-6500A conductivity meter, BELL Analytical Instruments Co., Ltd., Dalian, China)	[24]
Total carbon (TC) and total nitrogen (TN)	Combustion method (Vario ELIII Elemental analyzer, Elementar Company, Hanau, Germany)	[24]
Alkaline-dissolved nitrogen (AN)	Alkaline dissolution and diffusion method (Titrette titrator, Brand Company, Wertheim, Germany)	[24]
Total phosphorus (TP)	Sodium hydroxide fusion and molybdenum antimony colorimetric method (UV-1900i Ultraviolet-visible Spectrophotometer, Shimadzu (Shanghai) Laboratory Equipment Co., Ltd., Shanghai, China)	[24]
Available phosphorus (AP)	Sodium bicarbonate leaching and molybdenum antimony colorimetric method (UV-1900i Ultraviolet-visible Spectrophotometer, Shimadzu (Shanghai) Laboratory Equipment Co., Ltd., Shanghai, China)	[24]
Total potassium (TK) and available potassium (AK)	Flame photometry (FP6410 Flame Photometer, Shanghai Instrument & Electrical Analysis Instrument Co., Ltd., Shanghai, China)	[24]
Soil bulk density (SBD) and soil water content (SWC)	Ring knife method (Electric heating constant temperature vacuum drying oven, Liangchuang Intelligent Technology (Chongqing) Co., Ltd., Chongqing, China)	[24]
Soil organic matter (SOM)	Potassium dichromate-concentrated sulfuric acid external heating method (Titrette titrator, Brand Company, Wertheim, Germany)	[24]
Urease (URE)	Indophenolic acid colorimetric method (UV-1900i Ultraviolet-visible Spectrophotometer, Shimadzu (Shanghai) Laboratory Equipment Co., Ltd., Shanghai, China)	[25]
Alkaline phosphatase (ALP)	Disodium phenyl phosphate colorimetric method (UV-1900i Ultraviolet-visible Spectrophotometer, Shimadzu (Shanghai) Laboratory Equipment Co., Ltd., Shanghai, China)	[26]
Sucrase (SUC)	3,5-Dinitrosalicylic acid colorimetric method (UV-1900i Ultraviolet-visible Spectrophotometer, Shimadzu (Shanghai) Laboratory Equipment Co., Ltd., Shanghai, China)	[27]
Catalase (CAT)	Potassium permanganate titration method (UV-1900i Ultraviolet-visible Spectrophotometer, Shimadzu (Shanghai) Laboratory Equipment Co., Ltd., Shanghai, China)	[28]

Note: Soil particles were classified as clay (<0.002 mm), silt (0.02–0.05 mm), and sand (>0.05 mm) based on American standards.

## Data Availability

The original contributions presented in the study are included in the article. Further inquiries can be directed to the corresponding authors.

## Abbreviations

The following abbreviations are used in this manuscript:

WLYY00	*Salix cheilophila* + *Populus simonii* plantation
WL	*Salix cheilophila* plantation
YY	*Populus simonii* plantation
NT00	*Caragana korshinskii* plantation
LD	Bare land of flowing dune
